# Continuous Spinal Anesthesia Technique After Accidental Dural Puncture

**DOI:** 10.7759/cureus.29046

**Published:** 2022-09-11

**Authors:** Soumya Matturu, Amol Singam, Sheetal Madavi, Neeta Verma

**Affiliations:** 1 Department of Anaesthesiology, Jawaharlal Nehru Medical College,Datta Meghe Institute of Medical Sciences (Deemed to be University), Wardha, IND

**Keywords:** cauda equina syndrome, sensory block, spinal analgesia, post dural puncture headache, dural puncture

## Abstract

Continuous spinal anesthesia (CSA) is a mode of anesthesia and analgesia that has various therapeutic advantages. CSA allows the anesthesiologist to titrate tiny doses of a local anesthetic to achieve the desired degree of spinal anesthesia. The duration can be extended to accommodate the demands of the protracted operation. Due to a lack of equipment and financial restraints, particularly in resource-constrained areas, and worries of neurologic consequences such as cauda equina syndrome, CSA is yet to acquire general acceptability among anesthesiologists. In terms of postoperative pain management, CSA can be comparable to epidural analgesia and is considered far superior to abdominal wall blocks when correctly applied. Here we discuss a case wherein a standard epidural catheter in subarachnoid space was used to successfully perform an emergency exploratory laparotomy.

## Introduction

Epidural analgesia is one of the most common pain management methods; nevertheless, the placement of an epidural catheter might be confounded by unintentional dural puncture [1]. Following accidental dural puncture, either epidural can be placed in an intervertebral space proximal to the space where there was dura puncture, or converted to continuous spinal anesthesia (CSA), single-shot spinal anesthesia, or general anesthesia, or the treatment can be abandoned. CSA with an epidural catheter is an underutilized technique in modern anesthesia practice. CSA provides several therapeutic benefits for anesthesia and analgesia [2]. When compared to epidural or single-shot spinal procedures, the degree of sensory blockage may be titrated to the desired dermatomal level with remarkable accuracy, providing better management of the hemodynamic effects of sympathetic blockade associated with spinal anesthetics [3].

## Case presentation

A 49-year-old woman presented to the Emergency Medicine department at a rural hospital. She was apparently alright eight days when she developed abdominal discomfort, which was abrupt and gradual onset, intermittent, dull aching, and non-radiating. Other associated symptoms were constipation, stomach distention, and bilious vomiting. On general physical examination, the patient seemed to be a middle-aged, moderately built female with slight pallor, moderate dehydration, and tachypnea. The abdomen was bloated, and peritoneal symptoms were present. Complete blood count was normal with a hemoglobin level of 11 grams per deciliter. The patient’s consent was taken for general anesthesia with a neuraxial analgesic regimen as the mode of anesthesia after an abdominal ultrasound revealed dilated bowel loops, suggesting intestinal blockage requiring emergency surgery.

**Figure 1:**
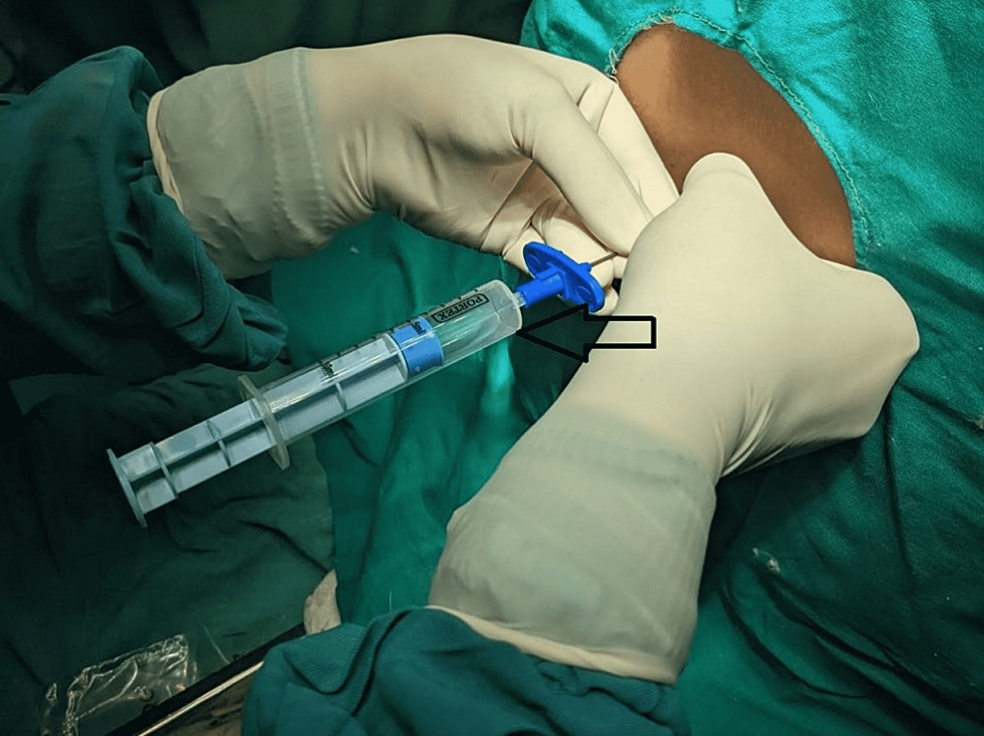
CSF in LOR syringe post-accidental dural puncture LOR, loss of resistance

In the pre-operative room, a full pre-anesthetic check-up was performed on an emergency basis. She belonged to the American Society of Anesthesiologists physical status of 1E (1 emergency). Pre-operative vitals were as follows: heart rate of 98 beats/minute and blood pressure of 100/60 millimeters of mercury. The patient was given ceftriaxone 2 grams and premedicated with ondansetron 4 milligrams after peripheral venous access was obtained, and all routine monitors such as pulse oximetry, noninvasive blood pressure monitoring, and 5-lead electrocardiography were attached and baseline vital were noted. A sterile environment was prepared, T10/11 (thoracic vertebrae 10-11) vertebral interspace was palpated, and an 18G Tuohy needle was inserted to find the epidural via midline approach following local infiltration with 3 ml of 2% lidocaine. At 5 cm, there was a decrease in resistance, and free-flowing cerebrospinal fluid (CSF) was noticed in the loss of resistance syringe (Figure 1). Following this, the needle was kept in situ. Verbal consent for CSA was taken, and the catheter was advanced into the intrathecal area with all resuscitative equipment and all emergency drugs ready. A total of 2 cubic centimeters of 0.5% isobaric bupivacaine was used to prime the 20G multiorifice catheter and filter, which was advanced 3 centimeters into the intrathecal area. During needle and catheter insertion, the patient did not experience any pain, and the catheter was fixed in place with clear sterile tape. To establish effective sensory blockage, a total of 10 mg of isobaric bupivacaine (2 ml) (given in 5 milligrams boluses) was administered (T4 to L2).

Modified Bromage scale was 1 to maintain appropriate sensory blockage during the procedure, and 10 micrograms of intrathecal dexmedetomidine and 5 mg of isobaric bupivacaine top-up were given. Following the top-up dosage of bupivacaine, the patient had an episode of hypotension (30% drop from baseline mean arterial pressure from 73 to 50), which was handled with 500 ml of Ringer's lactate to keep mean arterial pressure above 65 mmHg. During the surgery, the patient was given supplemental oxygen through nasal prongs and 1,800 ml of lactated Ringer’s solution.

Intraoperatively, she was diagnosed with a single cecal perforation, with fecal spillage. A double bowel diversion loop colostomy was performed after the solitary perforation detected at the caecum was closed. After surgery, the patient was sent to the surgical intensive care unit, where she was hemodynamically stable before being moved to the ward. Parenteral paracetamol 1 g eight hours a day was used to control postoperative discomfort, and the CSA catheter was withdrawn after 24 hours. The numerical rating scale was used to measure postoperative pain, with a score of 0/10 at 12 hours, 2/10 at 24 hours, and 2/10 at 72 hours.

She was discharged from the hospital on the 15th postoperative day with no neurologic side effects. The anesthetic approach was approved by both the surgeons and the patient.

## Discussion

CSA was originally documented in surgical patients some 100 years ago [3,4]. Although underutilized, CSA is a well-established neuraxial blockade method due to the rapid onset of sensory blockade onset and hemodynamic stability CSA is an alternate anesthetic approach to a single-shot spinal anesthetics and epidural anesthesia [5]. CSA allows the anesthetist to titrate small doses of local anesthetics to reach the required level of spinal anesthesia, and the duration can be extended to meet the needs of prolonged surgery, resulting in less interference with the cardiovascular and respiratory systems [6]. CSA was once considered only for the geriatric population and patients with cardiac history who were planned for noncardiac surgeries, but in recent times it is more commonly used for lower limb, urology surgeries, more recently, and gynecological procedures including labor analgesia. Despite the compelling safety evidence, CSA has still not gained widespread acceptance among anesthesia providers due to a lack of equipment and economic constraints, particularly in resource-constrained settings, and fears of neurologic complications such as cauda equina syndrome [7-9]. The cauda equina syndrome was assumed to be caused by either local anesthetic maldistribution caused by sluggish injection through microcatheters or the administration of excessive quantities of local anesthetics [8-10].

One of the most common neurological adverse effects following an accidental dural puncture is post-dural puncture headache (PDPH) [11]. It has been established that the risk of PDPH reduced significantly when the catheter inserted is left in situ for 24 hours. The proposed mechanism for this is the catheter it works as a mechanical barrier, preventing CSF from leaking through the hole made in the dura, as well as causing early inflammation at the dural puncture site, reducing CSF leakage further [12].

## Conclusions

CSA is a well-established approach with a low failure rate that avoids the hazards of general anesthesia while maximizing the benefits of both epidural and single-shot spinal anesthesia, such as the ability to extend anesthesia duration for lengthier operations. It is not a difficult procedure as previously presumed by many.
